# Radical prostatectomies for treatment of prostate cancer: trends in a ten-year period in public health services in the city of São Paulo, Brazil

**DOI:** 10.31744/einstein_journal/2022AO0049

**Published:** 2022-11-18

**Authors:** Lucas Seiti Takemura, Álan Roger Gomes Barbosa, Breno Santos Amaral, Alvaro Alexandre Dias Bosco, Danilo Budib Lourenço, Marcelo Apezzato, João Arthur Brunhara Alves Barbosa, Eliney Ferreira Faria, Bianca Bianco, Gustavo Caserta Lemos, Arie Carneiro

**Affiliations:** 1 Hospital Israelita Albert Einstein São Paulo SP Brazil Hospital Israelita Albert Einstein, São Paulo, SP, Brazil.; 2 Hospital de Amor, Barretos Barretos SP Brazil Hospital de Amor, Barretos, SP, Brazil.

**Keywords:** Prostatic neoplasms, Prostatectomy, Public health

## Abstract

**Objective:**

To analyze the characteristics of public health services related to radical prostatectomy, according to hospital volume of surgeries and stratified as academic and non-academic centers.

**Methods:**

An ecological study was conducted using a database available in TabNet platform of the Unified Health System Department of Informatics. Number of surgeries, length of hospital stay, length of stay in intensive care unit, in-hospital mortality rate, and cost of hospitalization were evaluated. The hospitals were divided into three subgroups according to surgery volume (tercile), and results were compared. The same comparisons were made among academic and non-academic centers. We considered academic centers those providing Urology residency program.

**Results:**

A total of 11,259 radical prostatectomies were performed in the city of São Paulo between 2008 and 2018. We observed a significant trend of increase in radical prostatectomies for treating prostate cancer over the years (p=0.007). The length of stay in intensive care unit, and number of deaths were not statistically different among centers with diverse surgery volume, nor between academic and non-academic centers. However, length of hospital stay was significantly shorter in academic centers (p=0.043), while cost of hospitalization was significantly higher in high-volume center compared to low- (p<0.001) and intermediate-volume centers (p<0.001).

**Conclusion:**

Length of hospital stay for radical prostatectomies performed in public services in the city of São Paulo was shorter in academic centers, whereas hospitals with a high volume of surgeries showed greater cost of hospitalization.

## INTRODUCTION

Worldwide, an estimated 1.3 million new cases of prostate cancer occur every year; it ranks second as most commonly diagnosed cancer in males. According to Bray et al., Brazil has 84,992 new cases of prostate cancer per year, accounting for 15.2% of all new cases of cancer diagnosed. It is also the third cause of cancer mortality, with 16,730 deaths annually.^(1)^ Data from the National Cancer Institute (INCA - *Instituto Nacional de Câncer*) reported 65,840 new cases of prostate cancer in Brazil each year between 2020 and 2022.^(2)^

Despite controversies about decline of mortality rate due to prostate cancer and some data on reduced incidence of distant-staged disease in the last years due to prostate-specific antigen-based screening, it has been performed worldwide.^(3)^ This screening is widely available for its low cost and is provided by the Brazilian Public Unified Health System (SUS - *Sistema Único de Saúde*).

There are few options to treat localized prostate cancer, such as active surveillance, radical prostatectomy, and radiation therapy, and the final decision is based on the shared consent of both patient and physician.^(4)^ The literature on radical prostatectomies shows lower complication rates and higher oncological success rates have been observed among high-volume surgeons;^(5,6)^ moreover, there is a drop in positive tumor margins at high surgery volume centers.^(7)^ The challenge for patients seen at SUS services is to reach high-volume centers and have access to better outcomes.

## OBJECTIVE

To analyze the characteristics of public health services related to radical prostatectomy, according to hospital volume of surgeries and stratified as academic and nonacademic centers.

## METHODS

This is an ecological study that analyzed data available between 2008 and 2018, from the TabNet platform of the Unified Health System Department of Informatics (DATASUS - *Departamento de Informática do Sistema Único de Saúde do Brasil*, http://www2.datasus.gov.br), which provides open data on procedures performed through the Brazilian public health care system. Procedures codes used for this study were “radical prostatectomy” (code 04.09.03.003-1), “prostatectomy in oncology” (code 04.16.01.012-1) and “radical prostatectomy in oncology” (code 04.16.01.013-0).

The following information was extracted from the TabNet dataset regarding radical prostatectomies: total number of surgeries performed, length of hospital stay, length of stay in intensive care unit (ICU), in-hospital mortality rate, and cost of hospitalization. The data regarding costs presented in this study were the amount of budget sent by the government to the organization to treat this specific condition and not the actual cost of each patient treated. The costs were in BRL (Brazilian reals). The analysis was conducted between 2008 and 2018.

First, the public hospitals in the city of São Paulo were divided into three subgroups according to number of surgeries performed (tercile), and the results were compared. Then, the same hospitals were divided into academic and non-academic centers. Those providing urology residency program were considered as academic centers.

### Statistical analysis

Statistical analysis was performed using SPSS 13.0 (SPSS for Mac OS X, SPSS, Inc., Chicago, IL, USA). Data was tested for normality with Shapiro-Wilk test. Numerical data were presented as median and interquartile range (IQR) and comparisons used the Mann-Whitney U test or the Kruskal-Wallis H test, followed by bivariate *post-hoc* tests for pairwise comparisons when appropriate. Categorical data were analyzed using the *χ*^2^ test.

Prais-Winsten linear regression model was adopted to check the temporality pattern of radical prostatectomies to treat prostate cancer over the period 2008 to 2018, including the annual percentage change (APC) and 95% confidence interval (95%CI). Additionally, the Durbin-Watson test was used for the classification of time trend rates, such as increasing, decreasing, or stationary.^(8)^ Statistical significance for all analyses was set at p<0.05.

This study was approved by the Research Ethics Committee of *Hospital Israelita Albert Einstein* (HIAE), (CAAE: 17208019.0.0000.0071; #3.625.161).

## RESULTS

**Figure 1:**
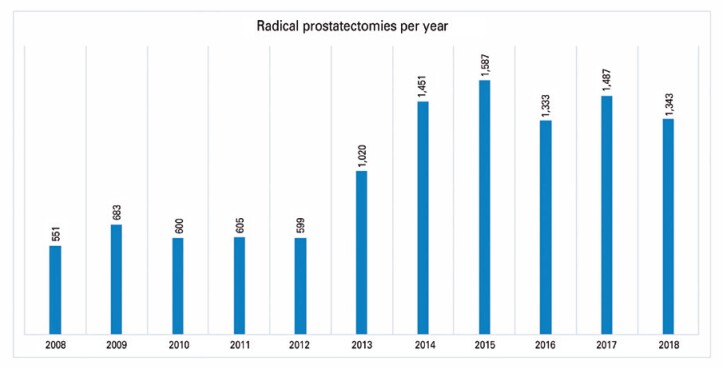
Radical prostatectomies performed per year between 2008 and 2018 in the city of São Paulo

A total of 11,259 radical prostatectomies were performed in the city of São Paulo between 2008 and 2018. The number of surgeries performed increased over the years, ranging from 551 radical prostatectomies in 2008 to 1,587 radical prostatectomies in 2015 (Figure 1).The procedures were performed in 32 institutions, of which 13 (40.6%) provide Urology residency programs. Regarding the volume of surgeries, the low volume group consisted of 11 hospitals that performed 1 to 6 radical prostatectomies each during the analyzed period; the intermediate group comprised 11 hospitals conducting 11 to 255 radical prostatectomies each; and the high volume group consisted of 10 hospitals that performed between 291 and 3,078 radical prostatectomies each. We observed a significant trend of increase in radical prostatectomies to treat prostate cancer over the years (APC 987.96; 95%CI: 337.32; 1638.60, p=0.007).

Comparisons of hospital volume of surgeries are shown in table 1. Length of hospital stay, length of stay at ICU, and number of deaths were not statistically different among low-, intermediate- and high-volume centers. The length of stay at ICU in all patients was <1 day. Finally, when the number of deaths was analyzed, high-volume hospitals had an in-hospital mortality rate of 0.002%, similar to the intermediate-volume group. There were no reported deaths in low-volume hospitals. However, the cost of hospitalization was significantly higher in high-volume centers compared to low-volume and intermediate-volume centers (both p<0.001).

**Table 1:** Length of hospital stay, length of stay at intensive care unit, cost of hospitalization, and in-hospital mortality rate of radical prostatectomies according to hospital volume

Variables	Hospital volume			p value
	Low	Intermediate	High	
Number of centers^#^, n (%)	11 (34.4)	11 (34.4)	10 (31.2)	-
Length of hospital stay (days)*	7.33 [2.75-9.38]	5.19 [4.42-6.82]	3.63 [3.13-3.96]	0.153^†^
Length of stay at ICU (days)*	0.0 [0.0-1.12]	0.09 [0.0-0.36]	0.24 [0.2-0.31]	0.532^†^
Cost of hospitalization (BRL)*	1.112,56 [1.022,64-1.439,86]	1.124,27 [1.064,27-2.204,72]	4.625,47 [4.013,34-4.882,88]	<0.001^†^
Number of deaths^#^, n (%)	0/37 (0.0)	2/852 (0.002)	27/1,037 (0.002)	0.94^‡^

* these variables are presented as median and interquartile range (IQR);

# these variables are presented as number/total and percentage (%);

† Kruskal-Wallis test with post-hoc test;

‡ χ^2^ test.

Cost of hospitalization showed significant difference between low- and high-volume (p<0.001) and intermediate- and high-volume (p<0.001) centers.

ICU: intensive care unit; BRL (Brazilian reals).

The division into academic and non-academic centers was as follows: there were 13 academic centers that performed between two and 3,078 radical prostatectomies, and 19 non-academic centers that performed between one and 1,091 radical prostatectomies during the analyzed period. Comparisons involving academic and non-academic centers are shown in table 2. The length of hospital stay was significantly higher at non-academic centers (p=0.043). There was no statistically significant difference when the length of stay at ICU was compared between academic and non-academic centers, and both had a mean length of stay of <1 day. The cost of hospitalization was not significant different between groups (p=0.234). In-hospital mortality rates in academic and non-academic centers were 0.24% and 0.29%, respectively (p=0.627).

**Table 2:** Length of hospital stay, length of stay at intensive care unit, cost of hospitalization, and in-hospital mortality rate of radical prostatectomies in academic and non-academic centers

Variables	Academic centers	Non-academic centers	p value
Number of Centers^#^, n (%)	13 (40.6)	19 (59.4)	-
Length of hospital stay* (days)	3.97 [3.16-4.83]	6.79 [3.49-9.12]	0.043^†^
ICU length of stay* (days)	0.21 [0.0-0.33]	0.2 [0.0-0.51]	0.906^†^
Cost of hospitalization* (BRL)	3.092,5 [1.235,38-4.314,07]	1.316,93 [1.077,15-2.996,91]	0.234^†^
Number of deaths^#^, n (%)	19/7,842 (0.24)	10/3,417 (0.29)	0.627^‡^

* these variables are presented as median and interquartile range (IQR);

# these variables are presented as number/total and percentage (%);

† Mann-Whitney U test;

‡ χ^2^ test.

ICU: intensive care unit; BRL (Brazilian Real).

## DISCUSSION

This is one of the first studies based on information extracted from a SUS database of a low-to-middle-income country comparing the outcomes of radical prostatectomies in different treatment scenarios. Our findings showed that length of hospital stay was lower in academic centers, while hospitals with a high volume of surgeries showed higher cost of hospitalization.

The Brazilian public health system is one of the most important and complex in the world, and it guarantees complete and indiscriminate free access to health care for the entire population. It is a national program organized by complexity of care. Brazilian citizens that demand treatment must first be evaluated by a general practitioner in primary healthcare units, generally in their neighborhood. Although most medical complaints of patients can be resolved in this first assessment, the individuals with diseases requiring specific treatments are referred to hospitals with higher grades of complexity. Unlike high-income countries, such as England,^(9)^ the Brazilian referral system has some limitations that hinder its optimal operation. First, SUS demands government investments greater than the actual amount provided by the authorities. Second, the majority of Brazilian inhabitants rely on SUS for medical treatment, and the population is growing every year. It is clear that health demand of the population is surpassing the current health system capacity of solving it,^(10)^ and the population is growing and aging, and more diagnosis of prostate cancer are made.

When we analyze the prostate cancer scenario in our country, we can observe a greater proportion of advanced cases due to problems in the screening program, which could impair and add complexity to treatment planning.^(11)^ It is also common to observe oncological patients being treated at lower complexity hospitals, probably due to the long waiting time in referral centers. This is considered a major issue for SUS since the centers with higher volume of surgeries tend to have better outcomes when compared to low-volume centers.^(5–7)^ It should be emphasized that this is an association and not a causal relationship. Most evidence is derived from observational studies, for it is not viable to carry out trials randomizing patients to be operated on at low- *versus* high-volume centers to compare the results. However, according to the Bradford Hill criteria, associations are more likely to be causal if they are strong, consistent, related to dose-response, temporally accurate (exposure before outcome), analogous to other causative associations, and based on a plausible and coherent mechanism.^(12)^ All these criteria were taken into consideration when a recent systematic review^(13)^ analyzed 49 publications about the topic, and most of them demonstrated higher-volume surgeries are associated with better outcomes, including reduced mortality, morbidity, postoperative complications, length of stay, readmission, and cost-associated factors. Our study, in accordance with these findings, also described high-volume centers had shorter length of hospital stay compared to intermediate-volume centers, although the difference was not statistically significant. However, the number of radical prostatectomies performed during the analyzed period was heterogeneous among centers; in that, each low-volume centers performed 1 to 6; each intermediate-volume centers, between 11 and 255; and each high-volume center between 291 and 3,078 procedures. In addition, our analysis of in-hospital mortality was impaired because there were no deaths reported in low-volume centers. This could be the result of underreporting or a statistical bias due to the small number of radical prostatectomies performed in these centers.

Another important aspect that influences in outcomes of radical prostatectomies is whether the surgeries were performed as open, laparoscopic, or robot-assisted approaches. Although a recent Cochrane review compared these three modalities and found no significant differences between them regarding oncological, urinary function, and sexual function outcomes; nonetheless, both laparoscopic and robotic radical prostatectomy resulted in statistically significant improvements in length of hospital stay and blood transfusion rates over open approach.^(14)^ Unfortunately, our study was based on a SUS database, and we had no access to patient medical history and details concerning the surgery.

Referral centers for prostate cancer treatment usually invest more to upgrade hospital facilities and acquire new technologies, focusing on minimally invasive procedures. Specialized staff and refined surgical technique based on a step-by-step procedure are keys to successful surgery. In Brazil, this scenario is usually related to academic centers, and urology residents are trained to perform radical prostatectomies according to these principles, always seeking the best practice possible. Our study demonstrated there was no significant difference in terms of in-hospital mortality between surgeries performed by urology residents (under supervision of a senior surgeon) compared to procedures performed only by urologists. In fact, the mean length of hospital stay was shorter in academic centers. Therefore, to improve the quality of treatment and simultaneously lower costs for SUS, the investments should be made in specialized centers throughout the country, preferably referring oncological patients to be treated there.

The National Cancer Prevention and Control Policy qualify healthcare centers as High Complexity Care Unit in Oncology (UNACON) or High Complexity Assistance Center in Oncology (CACON). Both centers are tertiary hospitals able to provide specialized care for diagnosis and treatment of cancer in Brazil; however, the former must provide care for the most prevalent cancers in the country, whereas the latter must provide care for all types of cancer.^(15)^ Cancer treatment is fully financed by the SUS and the procedures have a fixed reimbursement amount, regardless of size/type of healthcare center accredited by the SUS, including super-specialized procedures performed in oncology-qualified hospitals. Despite that, the DATASUS database covers only secondary and tertiary care, and some important pieces of information about patients’ demographics, pathological examinations or type of radical prostatectomy (open, videolaparoscopic or robot-assisted) are not provided. Moreover, the data available considered only details of one single hospitalization per patient; hence, costs involving future hospitalizations after discharges have not been considered in this study.

Finally, it is important to point out some limitations of our study. First, we analyzed only public hospitals in the city of São Paulo and all information collected was based on a regular reporting each institution provided to DATASUS. The data on outcomes were limited, and we had no access to patients’ medical history. Because our dataset was based on inpatient admissions, we were only able to assess in-hospital mortality, precluding comparisons with other studies that examined both in- and out-of-hospital mortality. Besides, a significant heterogeneity was observed regarding the number of surgeries performed among centers and this fact can hinder comparisons. Moreover, the costs presented in this study have some bias, since it is actually the amount granted by the government to the institutions to treat this specific condition, and not the actual cost of each patient treated. Therefore, high-volume hospitals had major costs compared to low- and intermediate-volume centers.

## CONCLUSION

In summary, outcomes of radical prostatectomies vary according to number of surgeries performed by hospitals and whether patient are treated in academic centers or not. Hospitals with a high volume of surgeries showed higher cost of hospitalization, and academic centers had shorter length of hospital stay.
